# Supplementary material for: The ATPase hCINAP regulates 18S rRNA processing and is essential for embryogenesis and tumour growth
Source: Nat Commun. 2016 Aug 1;7:12310. doi: 10.1038/ncomms12310 (PMC4974663; doi:10.1038/ncomms12310)
Supplement: Supplementary Information — Supplementary Figures 1-9 and Supplementary Table 1 [file ncomms12310-s1.pdf]

**a**

**b**

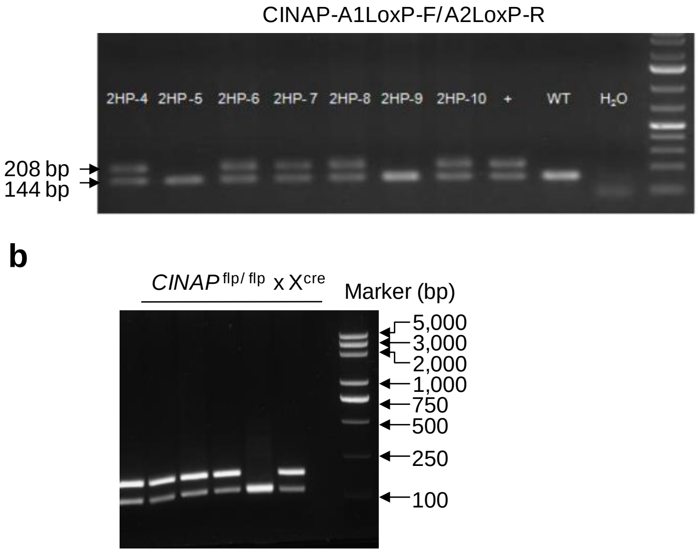


**Supplementary Figure 1** Deletion of the *CINAP* gene in mice. (**a**) Genotyping of F1 heterozygous *CINAP*$^{+/flp}$ mice. The chimeric mice were crossed with C57BL/6J mice to obtain F1 mice. PCR amplification of the wild-type CINAP allele and the mutated *CINAP*$^{flp/flp}$ allele yielded fragments of 144 bp and 208 bp, respectively. (**b**) PCR analysis of the deletion in *CINAP*$^{+/-}$ mice. Genomic DNA from the offspring of CMV-Cre and *CINAP*$^{flp/flp}$ mice was extracted. Deletion of the *CINAP* allele was detected with the indicated primers.

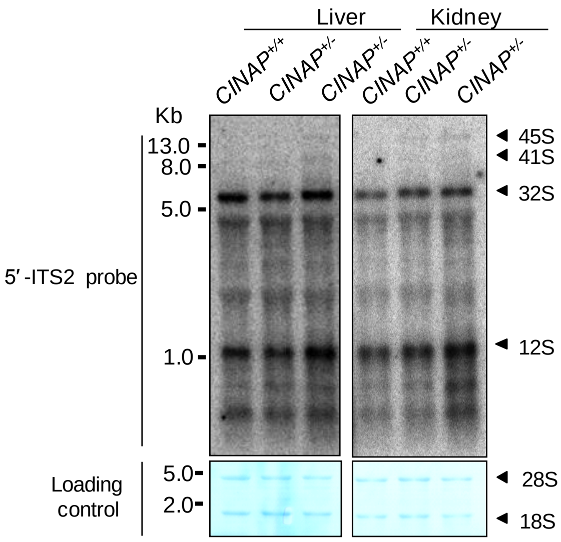


**Supplementary Figure 2.** CINAP reduction has no significant effect on the processing of 32S pre-rRNA. Total RNAs of liver and kidney tissue from *CINAP*$^{+/+}$ and *CINAP*$^{+/-}$ mice (female, 6 week of age, randomly selected) were exacted for northern blot analysis. The 5′-ITS2 probe showed equal steady-state levels of precursor RNAs in *CINAP* wild-type mice and heterozygotes. Methylene blue staining of 28S and 18S rRNA is shown as the loading control.

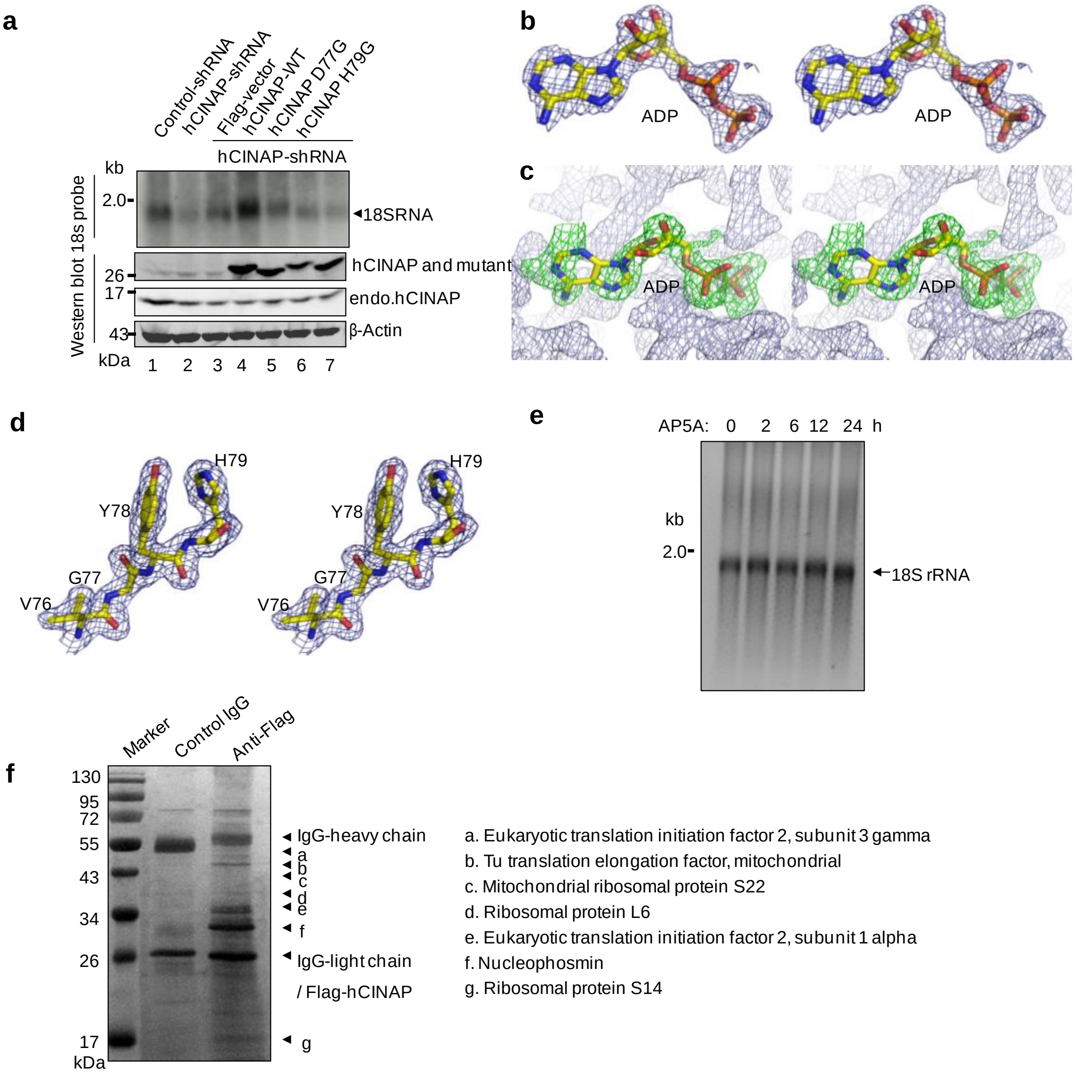
a
Control-shRNA
hCINAP-shRNA
Flag-vector
hCINAP-WT
hCINAP D77G
hCINAP H79G
hCINAP-shRNA
kb
2.0
18SRNA
Western blot 18s probe
26
hCINAP and mutant
17
endo.hCINAP
43
β-Actin
kDa 1 2 3 4 5 6 7
b
ADP
ADP
c
ADP
ADP
d
H79
Y78
G77
V76
H79
Y78
G77
V76
e
AP5A: 0 2 6 12 24 h
kb
2.0
18S rRNA
f
Marker
Control IgG
Anti-Flag
130
95
72
55
43
34
26
17
kDa
IgG-heavy chain
a
b
c
d
e
f
IgG-light chain
/ Flag-hCINAP
g
a. Eukaryotic translation initiation factor 2, subunit 3 gamma
b. Tu translation elongation factor, mitochondrial
c. Mitochondrial ribosomal protein S22
d. Ribosomal protein L6
e. Eukaryotic translation initiation factor 2, subunit 1 alpha
f. Nucleophosmin
g. Ribosomal protein S14

Continued

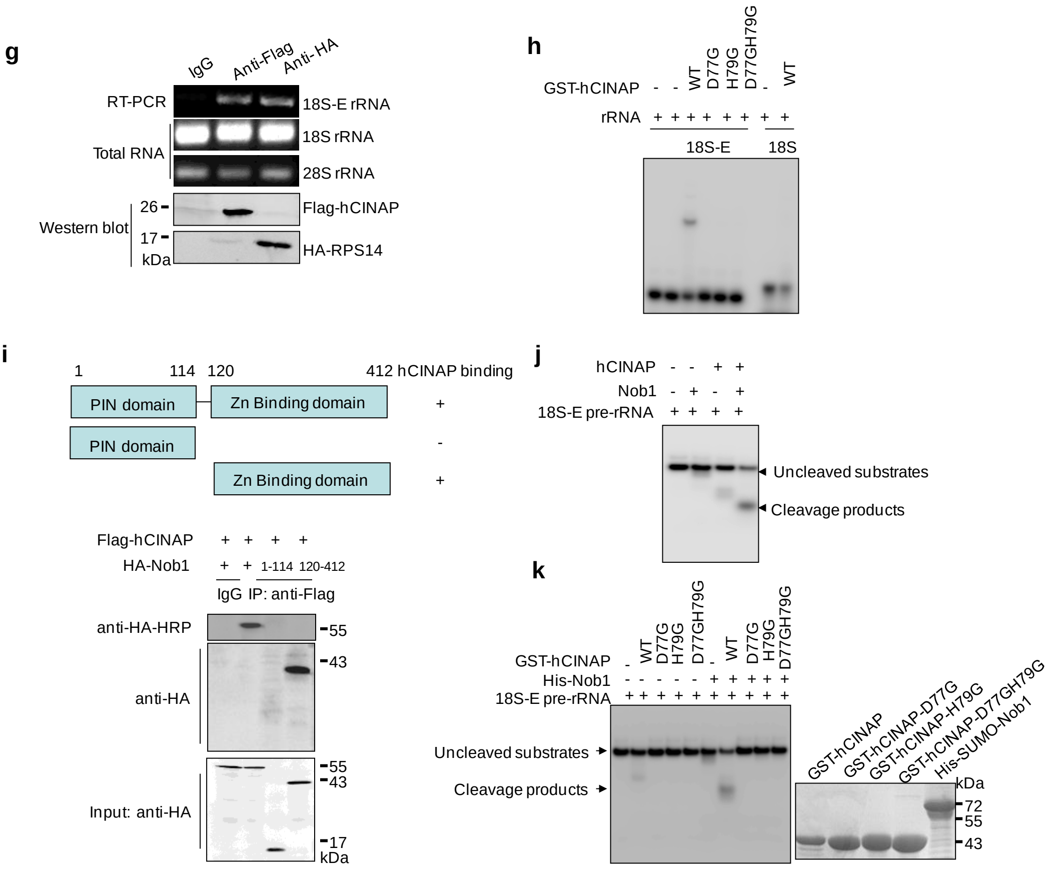


**Supplementary Figure 3**. ATPase activity of hCINAP is involved in 18S rRNA processing. (**a**) HeLa cells with depleted hCINAP were transfected with wild-type hCINAP or the ATPase-defective hCINAP-D77G, hCINAP-H79G, hCINAP-D77GH79G. 18S rRNA production was measured by northern blotting with 18S rRNA probe. (**b**) Stereo views of electron density maps (2$F_o$-$F_c$) at 1.4 ó level showing the presence of ADP molecule in the structure of hCINAP-D77G-ADP. (**c**) Stereo view of OMIT maps (green map, $F_o$-$F_c$) at 1.7 ó level showing the presence of ADP molecule in the structure of hCINAP-D77G-ADP. (**d**) Stereo views of electron density maps (2$F_o$-$F_c$) at 1.4 ó level around residues 76-79 in the structure of hCINAP-D77G-ADP. (**e**) HeLa cells were treated with AP5A (100 mM) for the

indicated periods. RNA was extracted and 18S rRNA production was detected by northern blot. (**f**) HEK 293T cell lysates were immunoprecipitated with anti-Flag antibody. Endogenous bound proteins were visualized on silver-stained SDS-PAGE gels. Specific bands were excised and subjected to mass spectrometry analysis. Parts of the identified proteins are listed. (**g**) 293T cells expressing Flag-vector or Flag-hCINAP were harvested and IP was carried out using anti-Flag antibody. The binding of hCINAP to 18S-E pre-rRNA was examined by PCR. (**h**) EMSA was performed using *in vitro* synthesized 18S-E pre-rRNA and purified wild-type hCINAP or the enzymatic mutant hCINAP-D77G and H79G. The 18S-E pre-rRNA was 5′-radiolabeled with $\gamma$-[$p^{32}$]-ATP and the signal was visualized by phosphor screen. (**i**) 293T cells were transfected with plasmids expressing hCINAP and the truncation of Nob1. 36 h after transfection, cells were harvested and subjected to Co-IP analysis. The interaction between hCINAP and truncated Nob1 was detected with the indicated antibody. (**j**) 5′-$^{32}$P-ATP labeled 18S-E pre-rRNA was incubated with purified hCINAP or Nob1. Pre-rRNA cleavage was visualized by autoradiography. (**k**) Wild-type hCINAP or ATPase-defective hCINAP was added to the cleavage system to evaluated 18S-E pre-rRNA processing by Nob1. The cleavage products were separated by denaturing 12% polyacrylamide/8 M urea gel and then the gel was exposed to phosphor screen. The signal was visualized by Typhoon TRIO Scanner (GE Healthcare). The purified hCINAP and Nob1 protein were examined by SDS-PAGE.

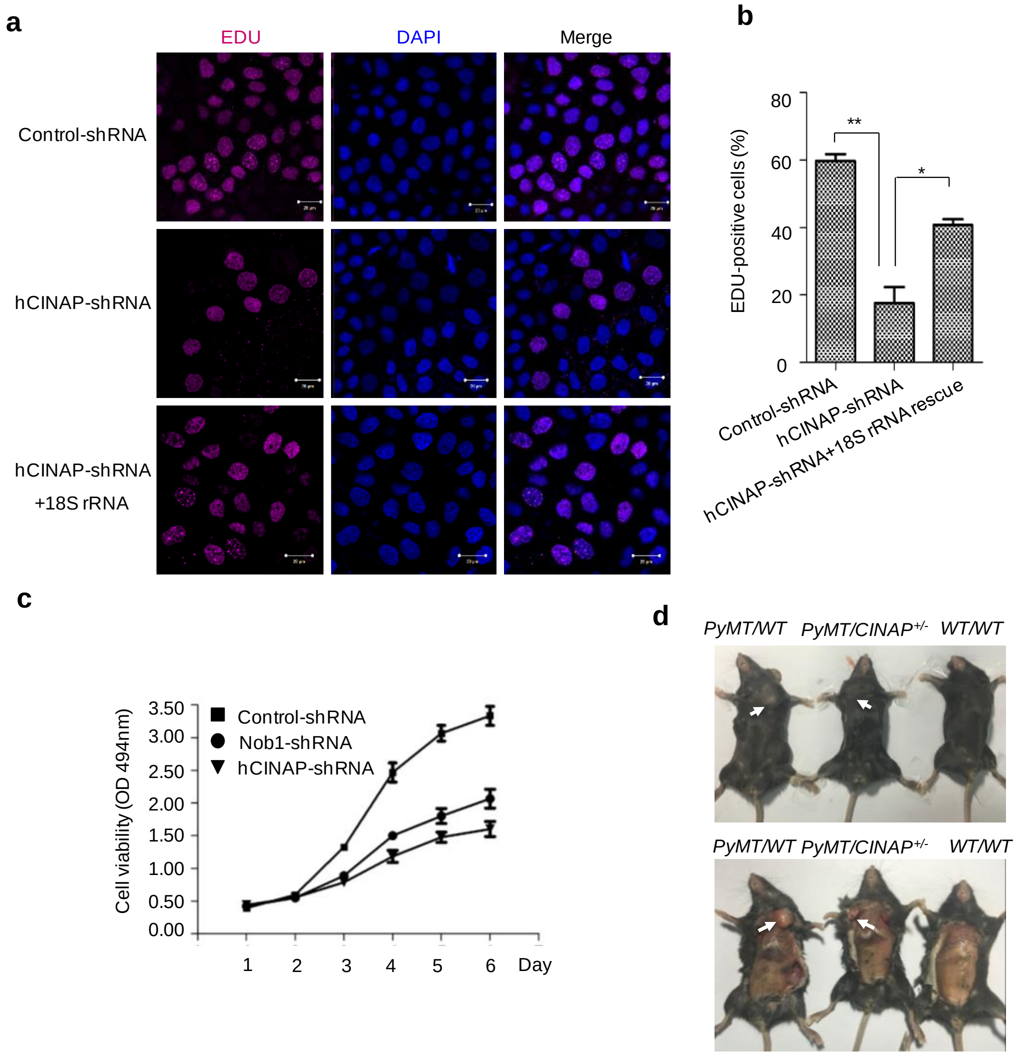


**Supplementary Figure 4.** hCINAP reduction inhibits cancer cell growth and tumor formation. (**a**) HeLa cells were transfected with lentivirus expressing control-shRNA or hCINAP-shRNA. The hCINAP-depleted cells were transfected with 18S rRNA and EDU (5 μM) was added to the culture medium to label the newly synthesized DNA for 48 h. Immunofluorescence assay was performed and the EDU signal was detected by Cy5 staining. The nuclei were visualized with DAPI. Scale bar, 20 μm. (**b**) The relative ratio of EDU-positive cells was obtained by randomly counting 10 images. Depletion of hCINAP decreased the cell proliferation and 18S rRNA rescue significantly recovered the cell growth inhibition by hCINAP reduction. **P*<0.05, ***P*<0.01. (**c**) MTT assay was performed using

U2OS cells with depleted hCINAP or Nob1. The abundance was measured at 494 nm. (**d**) Representative bright-field images of mammary adenocarcinoma from *PyMT/WT*, *PyMT/CINAP*$^{+/-}$ (n = 5) and *WT/WT* littermates (n=3). All the mice are female. Data of three mice at 120 days of age were shown. This experiment was performed according to the ethical guidance and approved by Peking University Laboratory Animal Center.

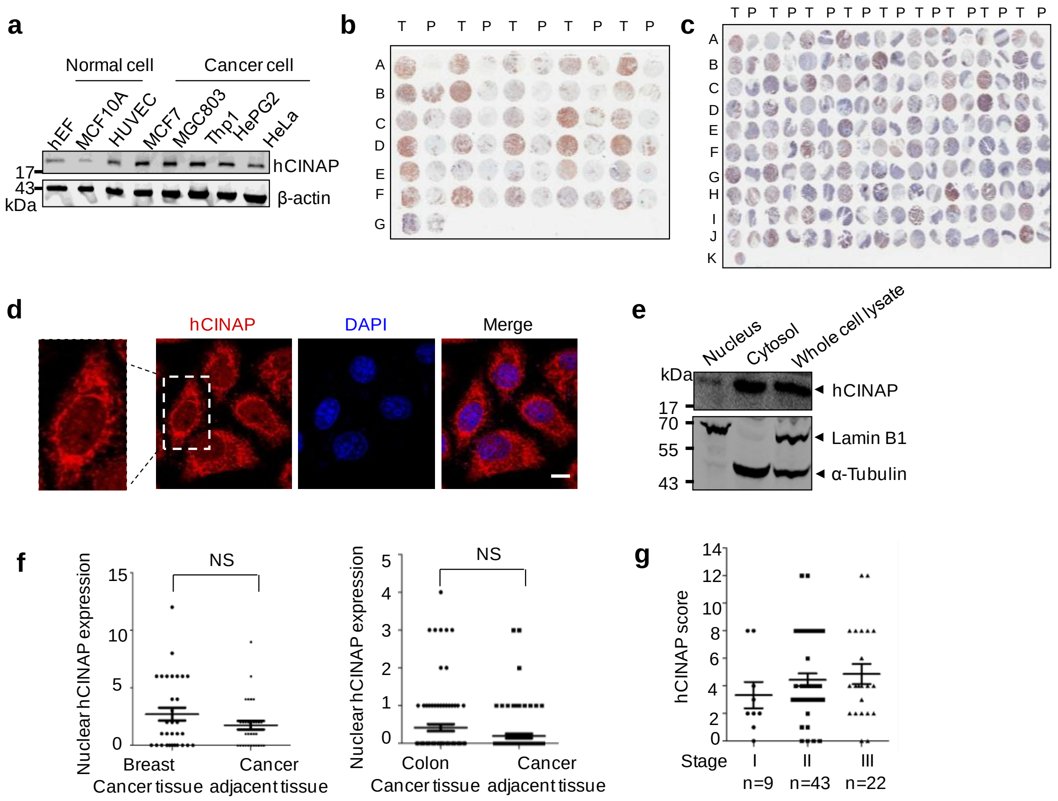


**Supplementary Figure 5.** hCINAP is highly expressed in human cancers. (**a**) Expression levels of hCINAP in a panel of human cancer cell lines and normal cells. Cells were collected and the level of hCINAP was evaluated with anti-hCINAP antibodies. (**b, c**) Full-view scans of hCINAP staining in breast cancer (**b**) and colorectal adenocarcinoma (**c**) tissue microarrays. T: tumor tissue; P: paracancerous tissue. (**d**) Immunofluorescence assay was performed to detect the subcellular localization of endogenous hCINAP with anti-hCINAP antibody. Scale bar, 10 μm. (**e**) Immunoblot analysis of cytoplasmic and nuclear fractions of endogenous hCINAP using anti-hCINAP antibody. Lamin B1 and α-Tubulin were used as the marker for the cytoplasm and nucleus, respectively. (**f**) Plots of the nuclear hCINAP expression in each pair of tissue samples. NS: no significance (Mann-Whitney U-test). (**g**) The expression levels of hCINAP in colorectal adenocarcinoma patient of disease stage I, II and III were analyzed. hCINAP expression slightly increased with the development of colon cancer.

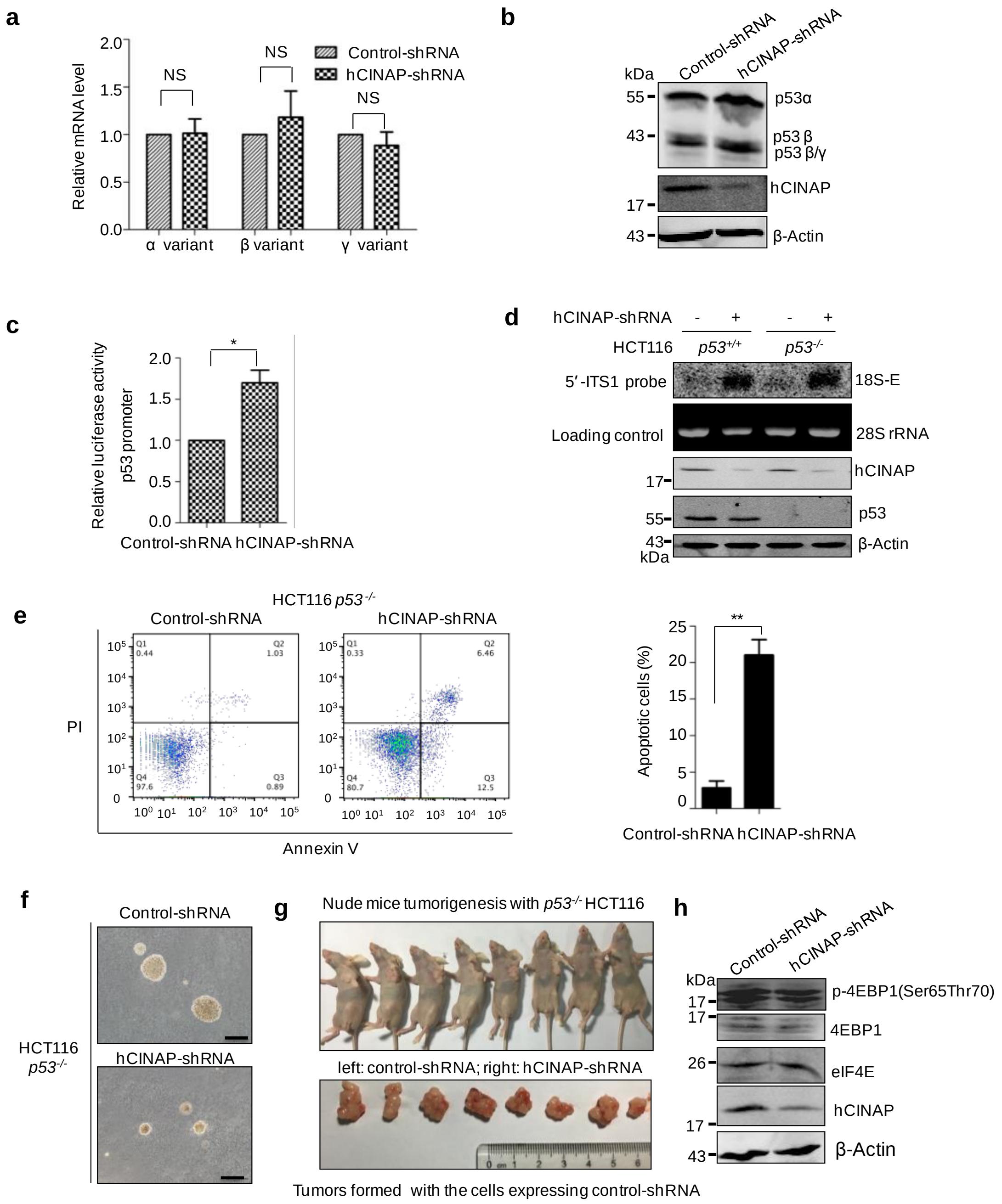


**Supplementary Figure 6.** The 18S rRNA processing defects and cell growth inhibition by hCINAP depletion is not dependent on p53. (**a**) Real-time qPCR assay was performed to evaluate the effect of hCINAP depletion on the splicing of the main isoforms of p53 including p53α, β and γ. (**b**) The protein abundance of each isoform of p53 was examined using p53 antibody (DO-1). (**c**) p53-Luciferase assay was carried out to quantify the p53 activity with

hCINAP depletion. *$P$<0.05 (Student's *t*-test). (**d**) Northern blot assay was performed using 5′ ITS1 probe to examine the effect of hCINAP depletion on 18S rRNA processing in $p53^{+/+}$ and $p53^{-/-}$ HCT116 cells. 28S rRNA was shown as the loading control. Depletion of hCINAP was examined by western blot using anti-hCINAP antibody. (**e**) Cell apoptosis rate of $p53^{-/-}$ HCT116 cells expressing control-shRNA or hCINAP-shRNA was evaluated by FACS analysis. Results were presented as mean ± s.d. *P*-values were determined by Student's *t*-test. ** $P < 0.01$. (**f**) Colony formation of $p53^{-/-}$ HCT116 cells harboring control-shRNA or hCINAP-shRNA. Cells were seeded in the soft agar and cultured for two weeks. The representative images of colonies formed by each type of cells were shown. Scale bar, 200 μm. (**g**) $p53^{-/-}$ HCT116 cells ($2\times10^6$) harboring control-shRNA or hCINAP-shRNA were injected subcutaneously into the flanks of nude mice (BALB/c, female, 5-6 weeks, n = 8) (Upper panel). Tumor formation was monitored once a week and after three weeks, mice were sacrificed and tumors were isolated (only control group generated tumors as shown in the left flank of mice. Lower panel). This assay was performed according to the ethical guidance and approved by Peking University Laboratory Animal Center. (**h**) The effects of hCINAP depletion on the abundance of eIF4E and the phosphorylation level of 4EBP1 were detected by western blot with the indicated antibodies.

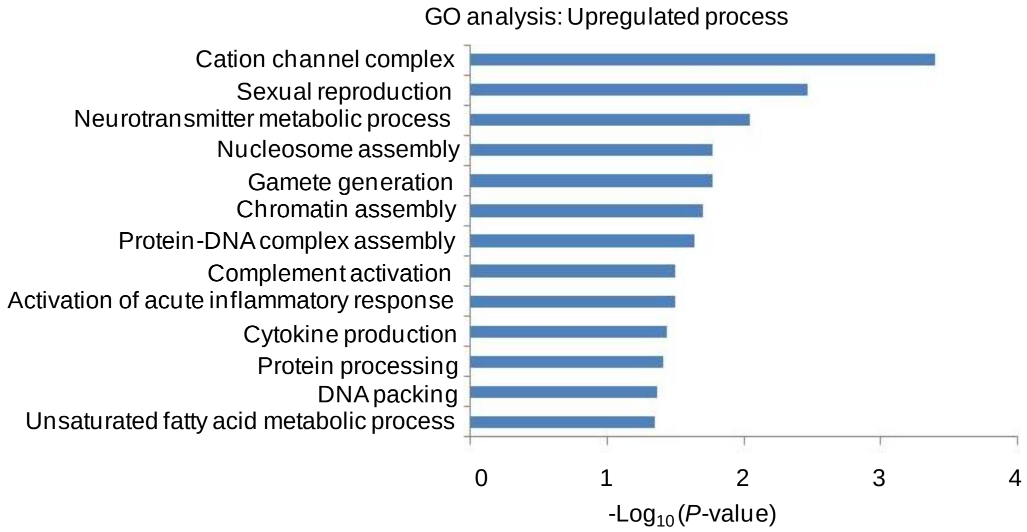


**Supplementary Figure 7.** Top 13 Gene Ontology (GO) term categories for mRNAs transcriptionally upregulated by overexpressed hCINAP. Bar-plot of the -log10 (*P* -value) for the significant ($P < 0.05$) GO terms from the biological process category.

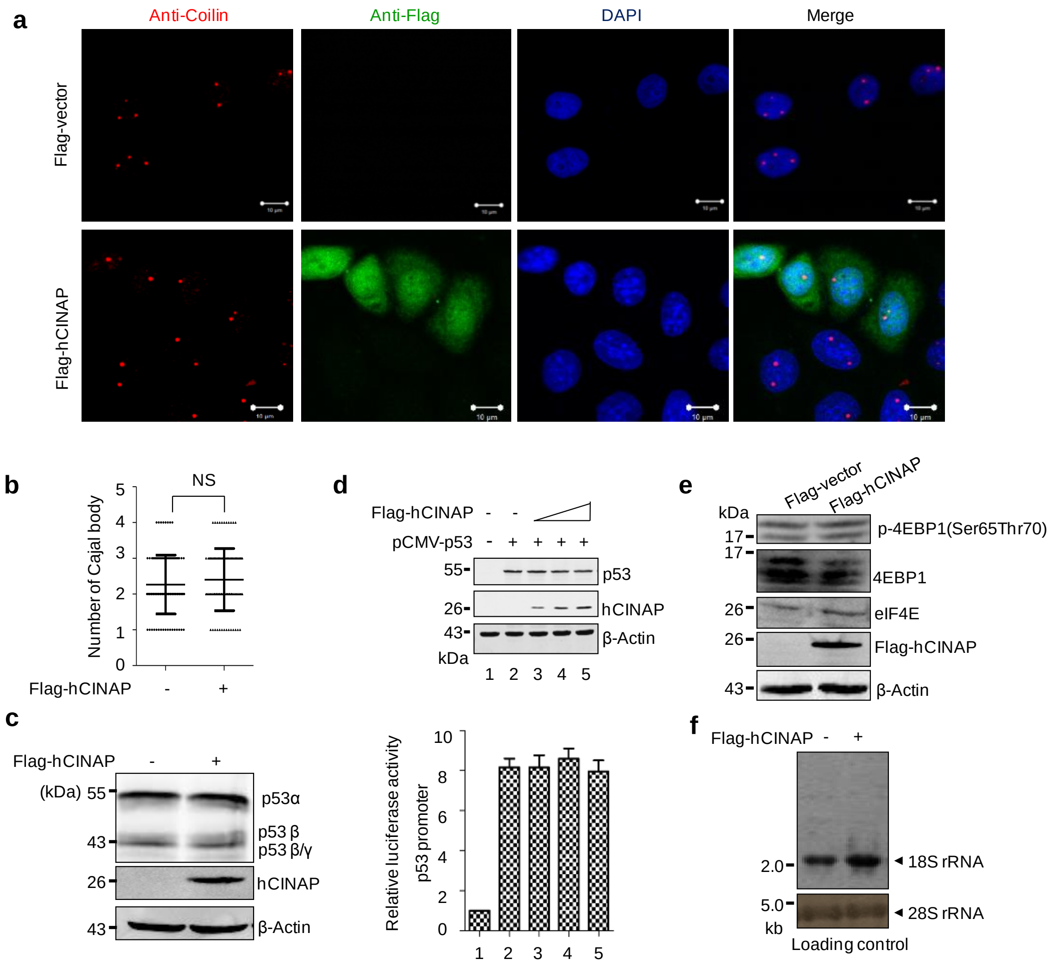


**Supplementary Figure 8.** The effects of hCINAP overexpression on Cajal body formation, p53 splicing and the expression of translation-associated factors. (**a**) Immunofluorescence assay was performed with anti-coilin antibody to detect Cajal body formation in HeLa cells expressing Flag-vector or Flag-hCINAP. Scale bar, 10 μm. (**b**) The number of Cajal body in the nucleus was counted from 100 nuclei resulting from more than 10 images from cell transfecting with Flag-vector or Flag-hCINAP, respectively. NS: no significance. (**c**) U2OS cells were transfected with plasmids expressing Flag-vector or Flag-hCINAP. The protein abundances of p53 isoforms (α, β and γ) were examined by western blot with anti-p53 antibody. (**d**) HCT116 cells were transfected with pCMV-p53 and an increasing amount of

Flag-hCINAP. The protein abundance of p53 was detected (upper) and the effect of hCINAP on p53 activity was examined by luciferase assay (lower). (**e**) The effects of hCINAP overexpression on the protein abundance of eIF4E and the phosphorylation level of 4EBP1 were detected with indicated antibodies. (**f**) HCT116 cells were transfected with Flag-vector or Flag-hCINAP for 36 h. Total RNA was extracted and northern blot was performed using 18S rRNA probe to detect 18S rRNA production. 28S rRNA was shown as the loading control.

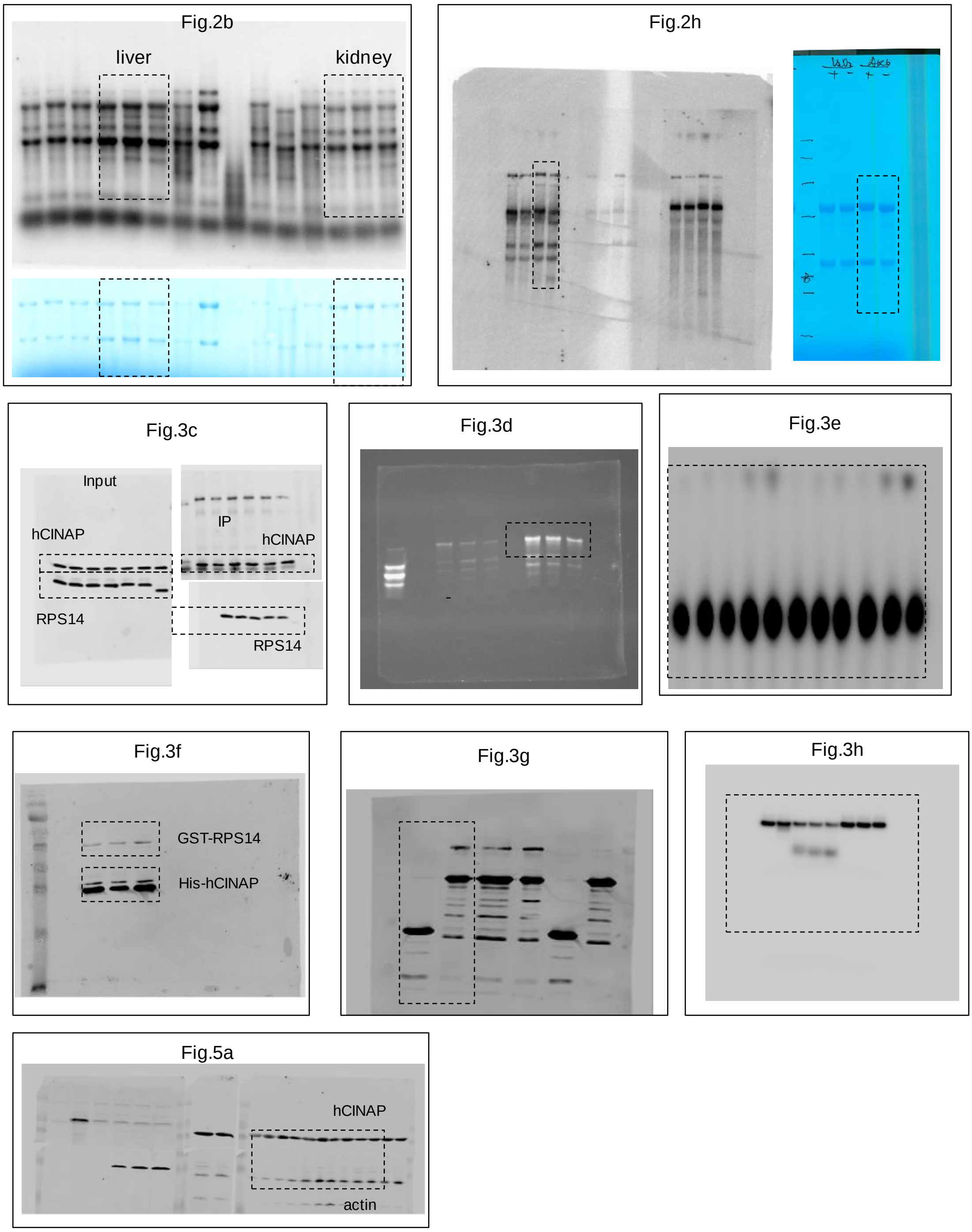


**Supplementary Figure 9.** Uncropped scans of the blots. Black boxes are the cropped bands in the indicated figures.

**Supplementary Table 1.** PCR strategy for detecting the genotypes of CINAP mice.

**a**

| Primer | Sequence | Tm/°C | Product size/bp |
|---|---|---|---|
| CINAP-A1 LoxP-F | AGGGTGGCACATCCTGTAATC | 58 | WT: 144<br>Mut:208 |
| CINAP-A2 LoxP-R | CGGCAACATGGCAACATAGC | 58 | |

**b**

| Genotype | A1 LoxP-F/A2 LoxP-R | | 3′loxP-F/3′loxP-R |
|---|---|---|---|
| | WT | Mut | |
| -/- | - | - | + |
| -/+ | + | - | + |
| +/+ | + | - | - |

(**a**) Primers used to detect the wild-type *CINAP* and $CINAP^{flp/flp}$ alleles. (**b**) PCR genotyping strategy of the $CINAP^{+/-}$ intercrossed offspring. The A1 LoxP-F/A2 LoxP-R primer pair was used to distinguish the wild-type and mutant CINAP alleles. Deletion of the CINAP allele was detected using the 3′loxP-F/3′loxP-R primer pair.
